# Resilin Distribution and Sexual Dimorphism in the Midge Antenna and Their Influence on Frequency Sensitivity

**DOI:** 10.3390/insects11080520

**Published:** 2020-08-11

**Authors:** Brian D. Saltin, Yoko Matsumura, Andrew Reid, James F. Windmill, Stanislav N. Gorb, Joseph C. Jackson

**Affiliations:** 1 Department of Electronic and Electrical Engineering, Centre for Ultrasonic Engineering, University of Strathclyde, 204 George Street, Glasgow G11 XW, UK; andrew.reid@strath.ac.uk (A.R.); james.windmill@strath.ac.uk (J.F.W.); joseph.jackson@strath.ac.uk (J.C.J.); 2 Department of Biomimetics, Hochschule Bremen—City University of Applied Sciences, Neustadtswall 30, D-28199 Bremen, Germany; 3 Department of Functional Morphology and Biomechanics, Zoological Institute of the University of Kiel, Am Botanischen Garten 1–9, D-24118 Kiel, Germany; yoko.matumura.hamupeni@gmail.com (Y.M.); sgorb@zoologie.uni-kiel.de (S.N.G.)

**Keywords:** *Chironomus riparius*, Diptera, insects, confocal laser scanning microscopy, finite element modelling, antennal hearing, biomechanics, multimodal sensor

## Abstract

**Simple Summary:**

The antennae of insects are multipurpose sensory organs that can detect chemicals, gravity, vibrations, and sound, among others. While such sensors are very specialized and adapted to their specific needs, the way the antenna itself is built has often been considered either uninteresting or unimportant. We used a laser to scan the antenna of the midge *Chironomus riparius*. Insect cuticle, if illuminated with laser light, reflects autofluorescent light, an emission that has long been known to indicate the material properties of the scanned cuticle sample. Rather than a simple beam-like structure of constant material stiffness, we saw bands of hard and soft material, distributed along the length of the antenna. We were able to computer-simulate the effect of this banded structure on the antenna’s resonant frequency and showed that it allows the beam to vibrate at different frequencies than would be expected only by its shape. This discovery will help us to better understand these animals’ biology and can inspire future biomimetic sensors for detecting sound or vibration.

**Abstract:**

Small-scale bioacoustic sensors, such as antennae in insects, are often considered, biomechanically, to be not much more than the sum of their basic geometric features. Therefore, little is known about the fine structure and material properties of these sensors—even less so about the degree to which the well-known sexual dimorphism of the insect antenna structure affects those properties. By using confocal laser scanning microscopy (CLSM), we determined material composition patterns and estimated distribution of stiffer and softer materials in the antennae of males and females of the non-biting midge *Chironomus riparius*. Using finite element modelling (FEM), we also have evidence that the differences in composition of these antennae can influence their mechanical responses. This study points to the possibility that modulating the elastic and viscoelastic properties along the length of the antennae can affect resonant characteristics beyond those expected of simple mass-on-a-spring systems—in this case, a simple banded structure can change the antennal frequency sensitivity. This constitutes a simple principle that, now demonstrated in another Dipteran group, could be widespread in insects to improve various passive and active sensory performances.

## 1. Introduction

Contrary to mosquitoes, whose bite is not only a nuisance but also a pathway for the transmission of disease, midges receive limited scientific attention. However, midges are numerous in both numbers of individuals and number of species [1] and have been shown to be ecologically important for aquatic and lotic systems [2,3], in terms of biomass and production [4]. The present study on the intricate antennal structure, especially of the male non-biting midge *Chironomus riparius*, aims to reveal some adaptations of these animals’ biology.

*Chironomus riparius* is a non-biting midge that, like many mosquitoes, displays swarming behaviour [5,6,7]. Since acoustic communications play an essential role in finding mating partners [5,7,8,9], it is reasonable to expect that there are similarities in the antennal form and hence properties in species of midges and mosquitoes whose mating behaviour includes swarming. Antennae are remarkable sense organs capable of responding to a variety of sense modalities all at once [10,11]. Known functions include senses of smell and gravity, windspeed detection and, in many species, acoustic perception, the latter postulated as long ago as the 19th century [12]. The flagellar nematoceran antenna is built by three elements: most proximally-the scapus, which is partially responsible for orienting the rest of the antenna, followed by the spherical pedicel, housing a Johnston’s organ, and most distally the flagellum, which in both sexes appears sub-divided. The number of sensory neurons in the pedicel of mosquitoes has been estimated to be around 16,000 [13]. Most neurons in the Johnston’s organ are thought to be involved with acoustic perception, although which ones remains a matter for debate [14].

In males the flagellum is densely covered by fibrillae (also known as setae). These are hair-like structures which are thought to improve sensory performance by increasing the drag of the antenna [8]. Antennae exhibit strong sexual dimorphism, and the female antennae have shorter and fewer fibrillae than the male antennae, which are often referred to as plumose. Despite the known complexity of these auditory systems, mechanical properties of insect sensory organs are often overlooked [15], with just one recent study on the antenna of swarming and non-swarming mosquitoes [16].

To provide another mechanical case study on dipteran antennae, we chose the swarming midge, *Chironomus riparius*. As in the previous study [16], which deployed state-of-the-art confocal laser scanning microscopy (CLSM), the present study presents morphology of the male and female antenna of *C. riparius* through observation of different autofluorescences of varying cuticle configurations. In turn, this study hints at a potential functional influence of the distribution of material composition on resonant tuning of the flagellum. During the last decade, inferring material properties in this way has become an established method [17,18,19,20,21,22,23]. CLSM furthermore has the advantage of allowing the imaging of whole structures with no loss of depth resolution, at higher resolutions than conventional light microscopy. In addition to this structural observation, finite element modelling (FEM) of the mechanical behaviour of the antennae with an elasticity distribution in accordance to the observed CLSM data (following the method of [24], see also [16]) shows the potential effect of element position on the mechanical sensitivity. Finally, we discuss the impact of sexual dimorphism of structures and material composition patterns on resonant tuning and its diversity among species in relation to their mating biology.

## 2. Material and Methods

### 2.1. Specimen Preparation

Prior to dissection, the animals were anaesthetised with CO_2_. Dissection was performed in phosphate buffer solution (PBS) (Carl Roth GmbH & Co KG, Karlsruhe, Germany). The specimens were briefly subjected to small amounts of Triton X-100 (Sigma-Aldrich Chemie GmbH, Steinheim, Germany), to remove air bubbles trapped on the surface by decreasing water surface tension. Triton X-100 then was washed repeatedly with the PBS to fully remove traces of Triton X-100. Microscopical observations were made after transfer of antennae or antennal fragments to glycerine (Carl Roth GmbH & Co. KG, Karlsruhe, Germany).

### 2.2. Confocal Laser Scanning Microscopy (CLSM)

To analyse local distributing patterns of material compositions within the antenna, we applied CLSM for insect cuticles according to the method established by Michels and Gorb [17]. This technique is successfully used in studies of a wide range of insect exoskeletons [17,23,25,26] including the antennae of mosquitos [16]. The method was applied here as described by Michels and Gorb [17] using a confocal laser scanning microscope, CLSM Zeiss LSM 700 (Carl Zeiss Microscopy GmbH, Jena, Germany). Samples were sequentially exposed to four stable solid-state lasers with wavelengths of 405, 488, 555, and 639 nm, and the excited autofluorescences were filtered with 420–480 nm band-pass and long-pass emission filters transmitting light with wavelengths ≥490, ≥560, and ≥640 nm, respectively. Then, we assigned blue, green, red, and (again) red to the micrographs captured using the filters, respectively, and superimposed them into a final image. To avoid oversaturation, the last two laser lines were combined into one “red” channel, each on 50% intensity. It has to be noted that colours are a product of the colour code applied to the material autofluorescence, and it does not reflect the natural appearance of the antennae. In superimposed images of insect exoskeleton parts, the colour code is as follows: (1) well-sclerotized structures are shown in red, (2) tough flexible cuticular structures are indicated in yellow-green, (3) relatively flexible parts containing a relatively high proportion of resilin appear light-blue and (4) resilin-dominated regions are visualized as deep-blue.

### 2.3. Finite Element Modelling (FEM)

Finite Element Modelling (FEM) with COMSOL 5.3a (Comsol Inc., Stockholm, Sweden) was conducted to determine the effects of the CLSM results on the mechanical behaviour of the antenna. As with the previous study [16]—where details of the modelling method were already described—the sole purpose of the present simulations is to show that banding and the location of said bands have the potential to influence the beam mechanics. Hence a simplification to a cylinder (10% shell volume) was deemed justifiable to limit computation time, while still encompassing all relevant features of the system.

Similarly, as opposed to stiffness, mass does not tend to be dramatically different between different types of specialised cuticle [27], and therefore it is assumed to be constant in the present simulations. The parts with higher stiffness were simulated with 5 GPa, medium-hard stiffness elements with about 0.5 GPa, and soft material is around 1 MPa (mimicking a typical value for resilin) [27,28,29].

While the effect of the articulation in the pedicel was not the subject of our study, an approximation was needed and this was achieved by modelling the entire articulations as a round disc at the base of the flagellum, whose flexibility was fixed [16]. For illustration of the basic cylindrical model, please refer to the inset in the FEM simulation figure.

## 3. Results

**Figure 1:**
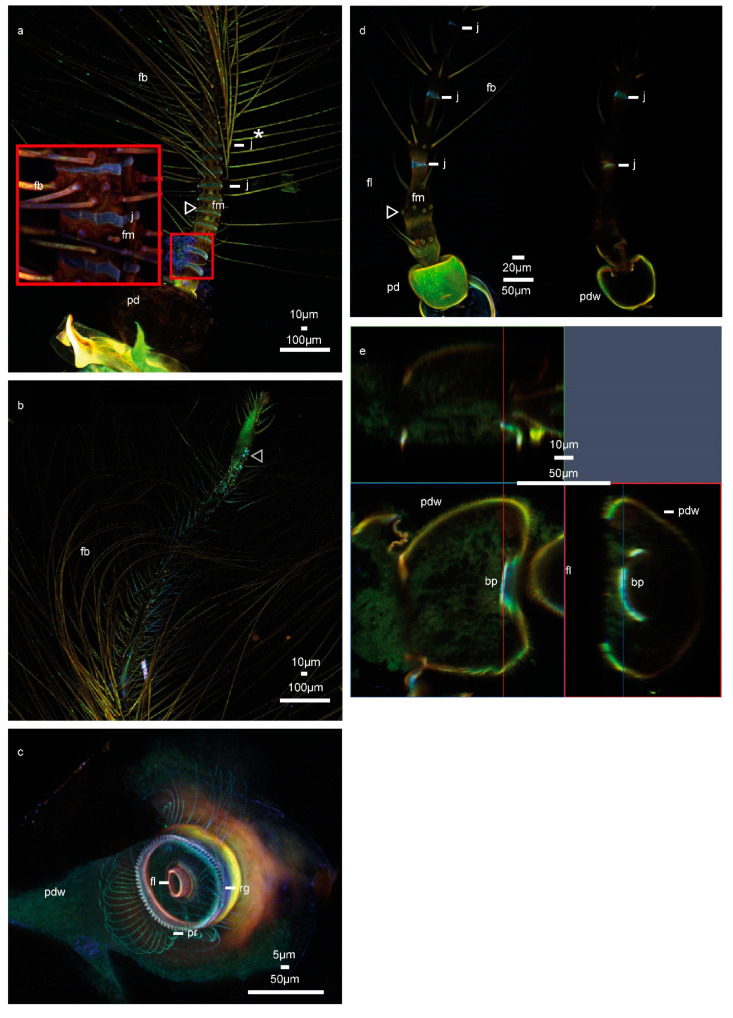
CLSM observations of the antenna of *C. riparius*. The colour code runs from blue colours for comparatively soft structures to increasingly stiff structures in red. (**a**) Maximum intensity projection of the male *C. riparius* antenna, pedicel and basal antenna part with magnified inset. White arrowhead: fibrillae sockets. (**b**) Maximum intensity projection of the male *C. riparius* antenna, tip region. Grey arrowhead: sensilla grooves (**c**) Maximum intensity projection of the male *C. riparius* pedicel, seen from inside. (**d**) Left: Maximum intensity projection of the female *C. riparius* antenna, right: cross-section. White arrowhead: fibrillae sockets (**e**) Optical cross-section of the female *C. riparius* pedicel. Abbreviations: bp: basal plate, fl: flagellum, fm: flagellomere, fb: fibrillae, j: joint, pd: pedicel, pdw: pedicel wall, pr: prongs, rg: ridge.

In the male *Chironomus riparius*, the pedicel is spherical and exhibits weak autofluorescence in comparison to the rest of the antenna (Figure 1a). The flagellum is composed of 11 units, called flagellomeres. With the exception of the most proximal flagellomere—whose flexible part might be hidden by the pedicel or be part of the articulation—the following ten flagellomeres consist of a basal flexible ring (blue) followed by a sclerotized (red) part, where, except for the 11th flagellomere (Figure 1a *), a circular crest of fibrillae emerges. In the most proximal 11th flagellomere (Figure 1b), fibrillae emerge in an apparently arbitrary pattern. The length of the flagellomeres decreases from the 2nd to 9th, and the lengths are approximately 20–30 µm. The flexible part of the proximal flagellomeres is similar in length to the sclerotized part. In more distal flagellomeres approaching the 10th flagellomere, the flexible part decreases in length to about half of the length of the sclerotized part. The sclerotized part, which is approximately similar in length, gradually loses the dominance of red autofluorescence. From the 5th or 6th flagellomeres onwards, their autofluorescence becomes entirely green (i.e., tough and flexible). The whole structure tapers continuously from the base to the 10th flagellomere—the diameter of the flagellomeres decreases from around 65 µm to 40 µm and continues to taper towards a pointed tip. After the 10th flexible ring (showing strongly blue autofluorescence), the antenna shows less intense autofluorescence until the tip. The fibrillae continuously become shorter along the flagellum up to the very short and irregular fibrillae at the tip. Along the flagellum, none of the fibrillae exhibits any strong autofluorescence (Figure 1a).

As in the other species previously investigated [16], all prongs are of the same diameter and show homogeneous green autofluorescence. The red-orange autofluorescence of the rim of the pedicel, already visible from the outside (Figure 1a), is also visible from the inside (Figure 1c). This indicates that the rim is relatively well sclerotized. The ridge, where the prongs attach, is deep-blue autofluorescent and possibly resilin-enriched, which is not encountered in any other species studied, while the prongs between attachment and flagellum appear to be of stiffer material indicated by reddish autofluorescence (Figure 1c).

In the female *C. riparius*, the pedicel is slightly rectangular in shape and exhibits comparatively strong green fluorescence (Figure 1d). The flagellum is composed of five flagellomeres, which are cylindrical but not constant in diameter within a flagellomere. All flagellomere have 6–8 separate long fibrillae emerging in a crest. There are rings of blue fluorescence (Figure 1d), which are likely resilin-enriched for flexibility. There is also another crest of shorter fibrillae present on each flagellomere. The long fibrillae emerge in one crest at the widest part of each flagellomere as it broadens, before the flagellomere tapers again. The bottom of each flagellomere, with diameters of 40 to 50 µm, tapers to about half this width (Figure 1c,d).

In the first and second flagellomere of *C. riparius*, fibrillae sockets of the fibrillae crest are apparent and are distinctly more orange/red than its remainder. To the right in Figure 1d, the optical section shows the articulation of the flagellum: no further internal details are visible. In comparison to the male and to the other species, the articulation is more flattened than domed (Figure 1d). An optical section (Figure 1e) of the pedicel shows a rather flexible soft articulation with a central blue area (Figure 1e). The pedicel as such is more fluorescent than the flagellum.

The male pedicel (Figure 1a,c), described in detail above, has a more detailed substructure than the female pedicel (Figure 1d,e). The female pedicel, described in detail above (Figure 1d,e), is in general more angular and less spherical, and the articulation of the flagellum is rather flat. There are clear differences in the flagellum’s subdivision in to flagellomeres and material distribution along the flagellum between sexes. Female antennae have five flagellomere, in contrast to the eleven flagellomere of the males. In both sexes, the hard parts of the flagellomeres are separated by blue-fluorescent joints. Furthermore, the female antenna is less covered by fibrillae (Figure 1a,d), which in both sexes similarly show relatively weak autofluorescences. The female does not exhibit the characteristic short intervals among green, red-orange, and light blue bands observed in the first ten antennal flagellomeres in males. Instead, the fewer flagellomeres are more evenly spaced out along the whole length of the female antennae.

**Figure 2:**
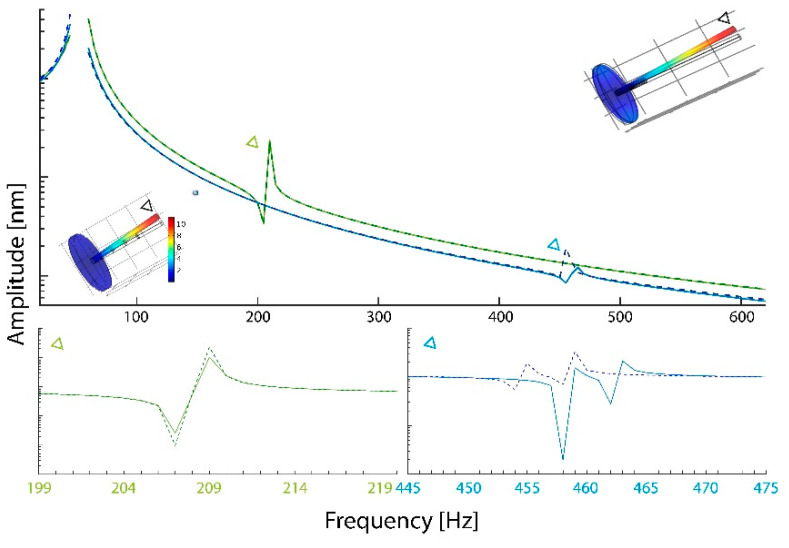
Simulation results for models of uniform and structured female and male *C. riparius* antenna in 5 Hz steps between 20 and 620 Hz. The figure includes for illustrative purposes the simulated female and male model (left and right, respectively). In each model the point (node), whose displacement is shown in the figure, is marked with a black triangle. The displacement is codified as gradient from low (dark blue) to high (red). Indicated in green (female) and blue (male), triangles point to the frequency of the strongest mechanical response. The figures underneath show the zoomed-in response for the female (left, green) and the male (right, blue). In each panel the comparison between a uniform beam of the sex-specific dimension depicted as solid line and the more natural situation of a substructured flagellum-beam depicted as dashed line, also in the sex-specific dimension. The used distribution of substructure is deduced from the antenna CLSM images Figure 1a,b (male) and Figure 1d (female). Simulating an impinging sound field, load was applied perpendicular to the beam axis in the +X direction on all but the lowest element.

Based on the observed flagellomere distribution and material distributions, FEM simulations of the mechanical response of a beam structure with and without the revealed substructure were performed (Figure 2). For the shorter female structure (green lines in Figure 2), no effect of the banded structure on the resonant frequency is seen. For the male antenna (blue lines in Figure 2) a small downwards shift of 4 Hz between the uniform structure (continuous light-blue line) and the more realistic substructured beam (dashed, dark blue line) can be observed. In the lower right corner, a more detailed 1 Hz step simulation of the frequency range around the strongest response 445–475 Hz in males is shown.

## 4. Discussion

Our results show that the well-known sexual dimorphism of dipteran antennae goes further than morphological structure alone, and in midge antennae also includes differences in material elasticity. Like in mosquitoes [16], material composition of the antennae is not homogeneous along the flagellum, but instead comprises hard and soft elements. Taken together with the structural complexity of the antenna in mosquitoes [16] and stick insects [30], it is becoming more evident that the structure and especially material and composition of insect antennae is much more complex as previously thought. Despite the statement that material properties of insect sensors are largely overlooked made as early as 2009 by Sane and McHenry [15], only limited research has been conducted to amend this lack of understanding. A lot of questions remain open and there is much potential for future research given the vast diversity of insect antennae not yet sampled. To our knowledge, none of the hitherto investigated species here and in our previous study [16] closely resemble each other regarding material distribution irrespective of their mating ecology. This indicates that further research will be necessary to better understand the various factors influencing antenna morphology. Given the different sensory functions of insect antennae that include, but are by far not limited to, olfaction, tactile sensation, and hearing, it is clear that the structure balances various trade-offs and functional constraints. One example of intricate structures of unknown function is the rapid sequence of flexible and sclerotized material at the base of the male flagellum of *C. riparius*.

Pedicels have a large variation of autofluorescence intensity and are largest in *C. riparius* females. In male *C. riparius,* a hard area on the distal ridge, where the flagellum emerges from the pedicel, is most prominently visible. The two important messages regarding the prongs are as follows. First, the prongs are neither particularly flexible nor stiff and are all amongst each other consistent in their autofluorescence within an individual animal. Secondly, judging from our CLSM images, they seem rather similar in dimensions. The uniformity of the prongs in their stiffness and dimension underpins previous assumptions by Avitabile et al. [31] that the prongs act more or less as rigid-body extensions of the flagellum. The flagellum, however, is by virtue of stiffness variation, shown by our study, potentially acts in a more complex manner than simply rigid beam of uniform stiffness. Similar to our study on mosquito antennae [16], we confirmed here the presence of variation and increased small-scale complexity of the dipteran antenna.

While the degree of effect remains under dispute, the direct fitness improvement of traits involved in sexual selection is not [32,33,34]. An impact of these differences on mating behaviour seems likely given the combination of the following three points: (1) certain mosquitoes (7–9) and at least some midges [5] respond to acoustic stimuli; (2) their antennae clearly show a well-known structural sexual dimorphism (e.g., [35]), and as demonstrated here also a dimorphism in material composition; (3) considerations by Loudon [36] heavily imply the importance of getting the flexural stiffness of antenna right for any given insect. This means that while the function of the different banded structure between species [16] and sexes reported remains unclear for now, they will be meaningful for the behaviour and biology of those animals.

Possible reasons for these antennal observations are that a different stiffness will inadvertently correspond to a different resonant tuning for acoustic perception, or for reasons of static integrity of the antenna, or perhaps another behavioural or ecological aspect of these animals’ biology. Compared to results in mosquitoes [16], the effect might be smaller in male Chironomidae or different in principle—both hypotheses require further investigation. Whatever the ultimate reasons for the observed specialisation are, it is fairly clear that different specialisations of males and females might require strong tuning of their acoustic sensors (antennae), which is not understood yet, but this study shows further evidence for the presence of such a specialisation. A limitation of both these studies is the lack of direct correlation of CLSM-based autofluorescence analysis with mechanical measurements, which should be tackled in follow-up investigations.

## 5. Conclusions

We have demonstrated that the sexual dimorphism in the antenna of *Chironomus riparius* pertains beyond geometry to material composition. The antennae of both sexes balance a variety of functions. Hence it is difficult to decide—without further research—how much of the newly found complexity actually is adaptive to a given sensory function. While effects on resonant tuning in male midges are small compared to the hundreds of Hz shifts observed in mosquitoes [16], variation in stiffness can alter the antenna’s vibrational characteristics in different species. 

This result and other studies on the mechanics of antennae [16,30] as well as other appendages [26], underlines the necessity of a more holistic and realistic future approach not only but especially for modelling. That includes the hitherto unknown material complexity in these structures.

Future studies of insect antenna could include investigations on other species or be combined with direct mechanical measurements, such as bending and indentation tests, which would provide better understanding of their structure-function relationships. Such outcomes will improve the quality of simulation results, as we clearly see how the mechanical responses can deviate due to structural and material complexities so far observed. The importance of knowledge about material properties of insect cuticle for understanding functional mechanisms of different organs is huge [21,26,30,37,38,39,40,41] e.g., for robotics [40,41], and can be extended to the sensory structures [16,41,42,43,44,45]. This is not only a matter of academic interest but could also feature in the improvement of biomimetic sensory systems with wide applications. Rather than trying to find materials with a given Young’s modulus to satisfy a design constraint, stiffness can be altered through careful design of banding with standard materials.
